# Importance of detection of *Strongyloides stercoralis* DNA in fecal samples from patients with type 2 diabetes mellitus

**DOI:** 10.1016/j.clinsp.2022.100060

**Published:** 2022-07-11

**Authors:** Márcia Carolina Mazzaro, Émelin Alves dos Santos, Gessica Baptista de Melo, Priscila Duarte Marques, Laura Vilela Souza, Jefferson Elias-Oliveira, Bruna Campos da Silva, Ronaldo César Borges Gryschek, Fabiana Martins de Paula, Rosângela Maria Rodrigues

**Affiliations:** a Laboratório de Parasitologia, Universidade Federal de Jataí, Jataí, GO, Brazil; b Laboratório de Investigação Médica, Hospital de Clínicase Instituto de Medicina Tropical, Faculdade de Medicina, Universidade de São Paulo, Brazil

**Keywords:** Strongyloidiasis, Diabetes mellitus, Parasitological diagnostic, Molecular diagnostic

## Abstract

**Objective:**

The association between diabetes and *Strongyloides* infection remains controversial. This study aimed to detect *Strongyloides stercoralis* DNA in the feces of patients with Diabetes Mellitus type 2 (DM2).

**Methods:**

Fecal samples were analyzed via the Lutz, Rugai, and agar plate culture methods. PCR amplification was performed using two targets (PCR-genus and PCR-species) located on the *S. stercoralis* 18S ribosomal.

**Results:**

The positivity for *S. stercoralis* using parasitological methods was 1.1%. PCR-genus (14.13%) demonstrated a higher positivity than PCR-species (9.78%).

**Conclusion:**

The results confirm the greater positivity of the molecular diagnosis in relation to parasitological methods, reinforcing its use as an additional tool for the diagnosis of *S. stercoralis* infection in patients with DM2 living in endemic areas for this helminthiasis.

## HIGHLIGHTS

• Positivity for strongyloidiasis in coproscopic exam was low in diabetic patients.
• PCR is more sensitive for detecting *S. stercoralis* infection in diabetic patients.
• Molecular diagnosis is an important tool for the detection of *S. stercoralis.*

## Introduction

*Strongyloides stercoralis* infection is a neglected tropical disease^1^ that affects approximately 350 million people worldwide, particularly in tropical and subtropical regions.^2^^,^^3^ The ability of *S. stercoralis* to cause systemic infection is an important feature of this parasite that may lead to hyperinfection syndrome and disseminated strongyloidiasis with a mortality rate of up to 100%, especially in the presence of immunological impairment.^4^^,^^5^

Diabetes mellitus is a metabolic disease and a serious health problem. Type 2 Diabetes Mellitus (DM2) is the most common form in terms of the number of people affected, disability, and premature mortality.^6^ Evidence suggests that inadequate control of blood glucose levels in diabetic patients contributes to susceptibility to infections,^7^^,^^8^ including parasitic infections.^9^ However, the relationship between diabetes and *Strongyloides* infection remains controversial, with both positive^10^ and negative^11^ associations. Furthermore, the presence of clinical situations associated with immunosuppression, such as prolonged use of corticosteroids, can predispose individuals to the development of severe forms of *S. stercoralis* infections.^12^, ^13^, ^14^, ^15^

The definitive laboratory diagnosis of *S. stercoralis* infection is based on the detection of larvae in the feces by microscopy. However, confirmation of infection is difficult because of the small number of larvae released in one's feces, particularly in the case of chronic infections.^5^ Molecular diagnosis is considered highly sensitive compared to parasitological methods and has been used to detect *S. stercoralis* infection in stool samples.^16^^,^^17^ In the context of *S. stercoralis* infection and diabetes, to date, very little research attention has focused on PCR for specific DNA detection.^15^^,^^18^ Thus, the present study aimed to detect *S. stercoralis* DNA in the feces of patients with DM2.

## Methods

### Ethics statement

This study was approved by the Research Ethics Committee of the Federal University of Goiás, GO (protocol number 929.187/2015) and by Secretaria Municipal de Saúde de Jataí, GO. Informed consent was obtained from each patient before specimen collection.

### Study population

This study was conducted in the municipality of Jataí, Brazil, which is located in southwestern Goiás State, 327 km from Goiânia (the capital of Goiás State) and 535 km from Brasilia (the capital of Brazil). The Health Care Network has 16 family health teams, corresponding to a population coverage of approximately 61.4%. The municipality of Jataí has an estimated population of 102,065 inhabitants.

The study was conducted from January 2015 to December 2016 and included patients with DM2 from the Diabetes Education and Control Program treated at the basic health unit of the municipality of Jataí, Goiás State. Inclusion criteria included any sex, age ≥30 years, diagnosis of DM2, use of insulin > 5 years, blood tests within the last two years, glycated Hemoglobin (HbA1c) > 6.5%, and no use of anthelmintic drugs in the last six months. Sociodemographic, clinical, and laboratory data were analyzed. All results were reported to the patients.

### Parasitological diagnostic

Three fresh fecal samples were collected on alternate days from each individual and sent to the Laboratório de Parasitologia, Universidade Federal de Jataí, Goiás for processing and analysis. The samples were analyzed using the Lutz, Rugai, and agar plate culture methods. Aliquots of samples were immediately frozen at -20°C for molecular analysis.

### Molecular diagnostic

#### DNA extraction

The molecular analysis was carried out at Laboratório de Investigação Médica (LIM06) at the Hospital das Clínicas of the Universidade de São Paulo, São Paulo, Brazil. DNA was extracted using the QIAamp DNA Stool Mini Kit (Qiagen Hilden, Germany) according to the manufacturer's modified instructions. A pool of three fecal samples from each patient was prepared (∼600 mg), followed by washing with a 2% Polyvinylpolypyrrolidone (PVPP) solution (Sigma-Aldrich, San Luis, Missouri, USA) in phosphate buffer (0 0.01 M, Ph 7.2). The pellet was used for DNA extraction. The DNA was then quantified using a NanoDrop ND-100 UV-VIS V3.2.1 (NanoDrop Technologies, Wilmington, DE, USA).

#### Polymerase Chain Reaction (PCR)

PCR amplification was performed using two sets of primers located on the *S. stercoralis* 18S ribosomal: genus-specific (PCR-genus [392bp, forward 5′-AAAGATTAAGCCATGCATG-3′ and reverse 5′-GCCTGCTGCCTTCCTTGGA-3′])^19^ and species-specific (PCR-species [101bp, forward 5’-GAATTCCAAGTAAACGTAAGTCATTAGC-3’ and reverse 5′-TGCCTCTGGATATTGCTCAGTTC-3]).^17^

The PCR reaction tests were performed at a volume of 25 μL containing ∼50 ng μL^−1^ of DNA, 2.0 μg of BSA, 0.2 mM each of dNTP, 1.5 mM MgCl_2_, 2 pM of each primer, a 1 ×  PCR buffer, and 0.5 U Platinum® Taq DNA polymerase (Invitrogen™, Thermo Fisher Scientific Corporation, Waltham, MA, USA). Amplification cycles were composed of an initial denaturation at 94°C for 2 min, followed by 30 cycles of 94°C for 1 min (denaturation), 60°C for 1 min (annealing), 72°C for 1 min (extension), and 72°C for 2 min (final extension). PCR amplification was conducted using a Mastercycler EP Gradient S Thermocycler (Eppendorf, Hamburg, Germany). The products were separated by electrophoresis in 2% agarose gel containing SYBR Safe (Invitrogen™). Negative (PCR mix with no DNA template) and positive (DNA from the filariform larvae of *S. stercoralis* collected from positive agar plates) controls were included in each amplification run. PCR products with positive amplification for each target were submitted for sequencing, and the sequences obtained were evaluated using the Basic Local Alignment Search Tool (BLAST).

### Data analysis

Descriptive analyses, including mean, Standard Seviation (SD), and percentages, were used to analyze the data.

## Results

### Sociodemographic data and parasitological diagnosis

A total of 92 patients with DM2 with a mean age of 62.3 years (±10.4) were included. Of these, 57 (61.96%) were women and 35 (38.04%) were men. Most of the patients were retired and had completed elementary school (Table 1).

**Table 1:** Socio-demographic (gender, age, employment, and education level) data of diabetes mellitus type 2 patients included in the study (n = 92).

Variables		Frequency (%)
Gender	Male	35 (38.0%)
	Female	57 (62.0%)
Age (years)	30‒50	14 (15.2%)
	51‒70	57 (62.0%)
	> 70	21 (22.8%)
Employment	Employee	18 (19.6%)
	Unemployed	12 (13.0%)
	Retired	50 (54.3%)
	Housewife	3 (3.3%)
	Autonomous	2 (2.2%)
	Pensioner	7 (7.6%)
Educational level	No formal	18 (19.6%)
	Primary	64 (69.6%)
	High school	7 (7.6%)
	Tertiary	3 (3.3%)

Based on the parasitological results, positivity in association with DM2 was 32.61% (30/92) for parasites and intestinal commensals. Regarding helminths, only one case (1.1%) of *S. stercoralis* associated with DM2 was observed. In addition, the protozoans *Blastocystis* sp. (7.61%), *Entamoeba coli* (5.43%), *Entamoeba hartmanni* (4.35%), *Endolimax nana* (3.26%), *Entamoeba histolytica* (2.17%), and *Giardia lamblia* (1.1%) were observed.

### Molecular diagnostic of the *Strongyloides stercoralis* infection

**Figure 1:**
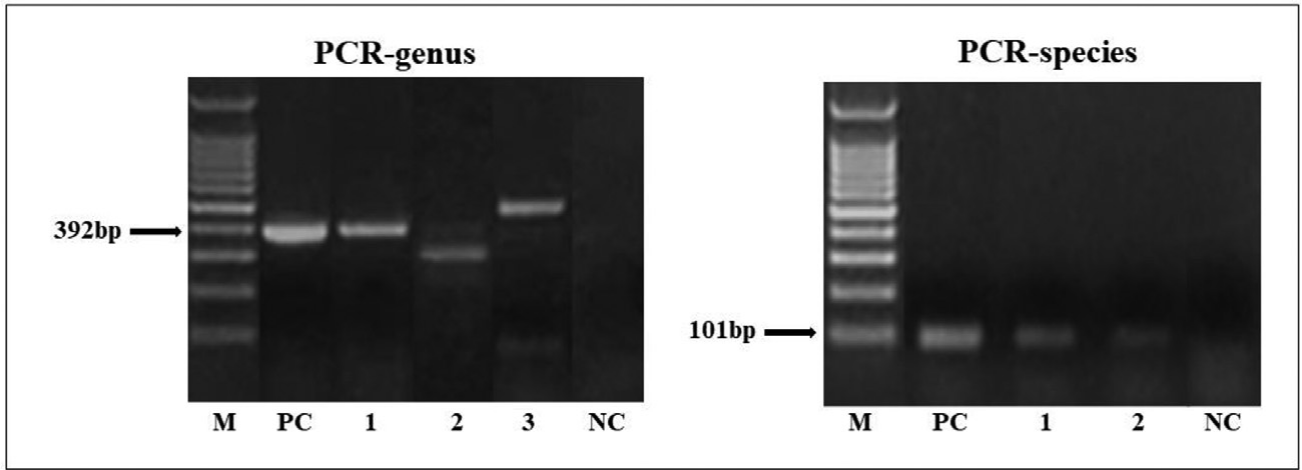
*Strongyloides* DNA amplification by PCR-genus (392 bp) and PCR-species (101 bp). M, 100 bp Molecular weight marker, NC, Negative Control (PCR mix with water); PC, Positive Controls (S. *stercoralis* DNA larvae), and DNA of fecal samples from diabetes mellitus type 2 (lane 1, 2 or 3).

In the PCR-genus, target fragment amplification (∼392 bp) was observed in 14.13% (13/92) of patients with DM2 (Fig. 1). The sequences obtained from the PCR genus were of low quality. It is important to note that of the eight patients with positive results in the PCR-genus test, four showed parasitological positivity for *Blastocystis* sp./*E. hartmanni* or *E. nana*, and three were positive for *E. nana* or/and *E. coli.* Non-specific amplification (∼400‒500 bp) were observed in 18 samples. Among these, *Blastocystis* sp. and amoebas were identified in six samples by parasitological methods.

The PCR species showed a positivity rate of 9.78% (9/92) in patients with DM2 (Fig. 1). All PCR-species products were of high quality and confirmed the identity of *S. stercoralis*. Six samples with positive amplification by PCR-species (∼101 bp) showed specific amplification by PCR-genus (∼392 bp). The patients with parasitological results that were positive for *S. stercoralis* were positive only in the PCR-species test.

The mean values of glycemia (228.1 mg/dL), HbA1c (9.6%), and eosinophils (244.4% and 3.3%) were higher in the PCR-species tests for DM2-positive patients (Table 2). Patients with positive amplification results are shown in Table 3. The mean HbA1c level was 9.2%, and the time since diabetes diagnosis ranged from 4 to 25 years (mean 12.7).

**Table 2:** Socio-demographic (gender, age) and laboratory (random blood glucose, HbA1c, eosinophil) data of diabetes mellitus type 2 patients (n = 92) according to the molecular results.

Variable		PCR-	PCR-genus+	PCR-species+
Gender (n = 92)	Male	26	7	2
	Female	44	6	7
Age (years), Means ± SD		63.2 ± 10.6	60.6 ± 8.3	58.3 ± 9.7
Random blood glucose (mg/dL), Means ± SD		211.4 ± 54.6	215.6 ± 51.7	228.1 ± 45.5
HbA1c (%), Means ± SD		9.0 ± 1.9	9.1 ± 1.8	9.6 ± 1.6
Eosinophil, count (%), Means ± SD		225.9 ± 224.0 (2.11 ± 3.14)	186.7±110.7 (2.6 ± 1.3)	244.4 ± 85.1 (3.3 ± 1.1)

**Table 3:** Characterization (Time DM2, random blood glucose, HbA1c, eosinophil, parasitological results) of diabetes mellitus type 2 patients according to the molecular results.

ID	Time DM2 (years)	Random blood glucose (mg/dL)	HbA1c (%)	Eosinophil		Parasitological results	Molecular results	
				Abs	(%)		PCR-genus	PCR-species
232	12	206	8.8	57	1	-	+	-
267	16	252	10.4	215	4	*E. coli; E. nana; E. hartmanni*	+	+
295	10	223	9.4	195	3	-	+	+
313	10	229	9.6	74	1	*G. lamblia*	-	+
467	12	157	7.0	380	4	*E. coli*	+	+
529	10	237	9.9	248	3	‒	-	+
535	25	260	10.7	229	3	*S. stercoralis*	-	+
587	15	189	8.2	55	1	*E. nana*	+	-
637	15	318	11.0	83	1	‒	+	-
639	12	312	12.5	268	4	‒	-	+
676	14	186	8.1	320	5	*E. coli*	+	+
847	10	194	8.4	101	2	‒	+	-
943	12	197	8.5	271	3	‒	+	+
1090	6	163	7.3	199	4	‒	+	-
1094	13	246	10.2	247	3	‒	+	-
1096	4	137	6.4	340	4	*B. hominis; E. hartmanni*	+	-
1098	20	235	9.8	237	3	‒	+	-

## Discussion

Despite decades of investigation, the association between diabetes and *Strongyloides* infection remains controversial.^10^^,^^11^^,^^20^, ^21^, ^22^ Notably, strongyloidiasis is a neglected tropical disease and detection tests used in primary health care generally have low sensitivity.^23^ In addition, severe forms of strongyloidiasis have been reported in patients with diabetes, particularly when they have a condition associated with immunosuppression.^13^^,^^14^

Several parasitological techniques have been used for the diagnosis of *S. stercoralis* infection;^4^^,^^5^ however, the positivity rate is usually low, which can lead to false-negative results. In the present study, a 1.03% positivity rate for *S. stercoralis* was detected using parasitological techniques. Similar results were reported in a study of parasite frequencies in individuals with type 1 and type 2 diabetes in the Federal District of Brazil.^24^ In a review study,^25^ Brazil was characterized as a hyperendemic area, with an occurrence of 5.5% for *S. stercoralis* infection and an estimated frequency of 6.6% in the Midwest region.

Analysis of three stool samples per individual via the Rugai and agar plate culture methods ‒ techniques indicated for the search for larvae, can increase the detection of infection^5^^,^^2^ and these methods were employed in the present study. However, parasitological methods may fail to detect *S. stercoralis*, particularly in patients with chronic asymptomatic infection or minimal symptoms.^5^ It is worth noting that the patients included in the present study had no gastrointestinal symptoms.

It is understood that problems related to the sensitivity of parasitological methods for the detection of *S. stercoralis* can be solved using molecular methods.^16^^,^^17^^,^^26^ The detection of *Strongyloides* DNA in fecal samples has been the objective of research by several groups,^27^^,^^28^ particularly in cases of immunocompromised patients.^29^ However, the molecular diagnosis of *S. stercoralis* infection has been only minimally explored in the context of diabetes. In two recent case reports,^15^^,^^18^ *S. stercoralis* infection in patients with diabetes was confirmed by PCR, suggesting a combination of parasitological and molecular methods for the diagnosis of helminthiasis.^30^

The present study is the first molecular analysis using two primers for the detection of specific *Strongyloides* DNA in fecal samples from patients with DM2. The positivity rates were 14.13% and 9.78% by PCR-genus and PCR-species, respectively. Regardless of the target, the positivity of PCR tests was higher than that of the parasitological methods, which has also been confirmed in other studies.^28^^,^^29^ The use of more sensitive methods to detect *S. stercoralis* infection in endemic areas such as Brazil can minimize the possible complications of severe strongyloidiasis in immunosuppressed patients.^4^^,^^15^

A fundamental step in the application of molecular methods is the choice of targets.^26^ The present results align with the data presented by Sitta et al.,^27^ who observed lower quality sequences obtained with the PCR-genus with *S. stercoralis* sequences present in the database. This can be explained by the amplification of different regions of the ribosomal gene, which is common in other organisms.^31^ Thus, the possibility of false positives cannot be ruled out, even with the visualization of amplification products with sizes similar to the target fragment, which supports the importance of sequencing.

Furthermore, PCR-species can act as an important tool in the molecular diagnosis of *S. stercoralis* infection, and the literature has indicated the high sensitivity and specificity of the species-specific primer.^16^^,^^17^^,^^27^ A study by Sitta et al.^27^ evaluating a panel of DNA obtained from fecal samples positive for *S. stercoralis*, positive for other parasitic infections, and negative, showed superior performance by the PCR-species versus PCR-genus. A species-specific primer was used in conventional PCR and real-time PCR for the detection of *S. stercoralis* DNA in fecal samples from transplant candidates, and the results showed good diagnostic performance.^29^ In the present study, all samples with positive amplification for PCR species were confirmed by sequencing to be *S. stercoralis.*

The potential limitations of this study include the small number of patients with diabetes analyzed and the absence of a control group, which could support the hypothesis of an association between *S. stercoralis* infection and DM2. However, the results reinforced the high sensitivity of molecular diagnosis in relation to parasitological in the detection of this helminth.

In conclusion, hyperinfection syndrome and dissemination of *Strongyloides* infection are associated with a high mortality rate, thus emphasizing the need for adequate screening tests to detect helminthiasis when a patient with diabetes has associated diseases that result in immunosuppression. Therefore, molecular methods can be considered an additional tool for the diagnosis of strongyloidiasis, particularly in patients with DM2 who live in areas in which *S. stercoralis* is endemic.

## Authors' contributions

MCM and GBM: Conceptualization, writing (original draft, review, and editing); EAS, LVS, JEO, and BCS: supervision, writing, and visualization; PDM, RCBG, and FMM: writing (draft, review, and editing); FMP and RMR: conceptualization, supervision, writing (review and editing), and visualization.

## Financial support

This work was supported by the researchers themselves.

## Declaration of Competing Interest

The authors declare no conflicts of interest.
